# Landless female peasants living in resettlement residential areas in China have poorer quality of life than males: results from a household study in the Yangtze River Delta region

**DOI:** 10.1186/1477-7525-12-71

**Published:** 2014-05-15

**Authors:** Ying Liang, Shuqin Li

**Affiliations:** 1 Department of Social Work and Social Policy, School of Social and Behavioral Sciences, Nanjing University, Nanjing 210023, Jiangsu province, People’s Republic of China; 2 School of Social and Behavioral Sciences, Nanjing University, Nanjing 210023, People’s Republic of China

**Keywords:** Landless female peasants, QOL, WHOQOL-BREF, China, Yangtze river delta, CFA, Gender differences

## Abstract

**Background:**

Urbanization has accelerated in China, and a large amount of arable land has been transformed into urban land. Moreover, the number of landless peasants has continually increased. Peasants lose not only their land, but also a series of rights and interests related with land. The problems of landless peasants have been long-standing; however, only a few studies have examined their health or quality of life (QOL). This paper assesses the QOL of landless peasants in the Yangtze River Delta (YRD) region, analyzes gender differences, and explores health inequity.

**Methods:**

Data are derived from household samples in six resettlement residential areas of three cities (Nanjing, Hangzhou, and Yangzhou) in the YRD region (N = 1,500; the effective rate = 82.4%). This study uses the short version of World Health Organization Quality of Life questionnaire (WHOQOL-BREF) scale to measure the QOL of landless peasants, and performs confirmatory factor analysis (CFA) and analyze gender differences in QOL on the basis of CFA.

**Results and conclusion:**

***First***, we use Analysis of Variance and Non-parametric Tests to test if the differences of mean value of testing generals have statistical significances. Results shows significant differences occur between the impacts of different genders on the four domains of QOL (physical health, psychological health, social relationships, and environment). The internal reliability of the WHOQOL-BREF scale is good (Cronbach’s alpha > 0.8), and the four domains of QOL are connected with each other. ***Second***, scores in each QOL domain are commonly low, whereas the scores of females are much lower, indicating a poorer QOL than that of males. ***Third***, results of the CFA of the QOL domains and their related observed variables indicate a good model fit. ***Fourth***, results imply that the order of importance of the four domains (psychological health (males = 26.74%, females = 27.17%); social relationships (males = 26.23%, females = 25.35%); environment (males = 25.70%, females = 24.40%); and physical health (males = 21.33%, females = 23.08%)) affecting QOL from high to low is the same for landless male and female peasants, whereas the proportion of importance is different between genders. The results highlight the importance of government intervention to improve the QOL of Chinese landless peasants, ultimately reducing health inequity.

## Introduction

Industrialization and urbanization have accelerated in China in recent years, and these trends have been accompanied by the increasing scale of arable land expropriated and the increasing number of landless peasants. The land of approximately 45 million peasants had been expropriated in rural China by 2007. Based on the growth rate and huge command of construction in the urbanization of China, the number of peasants whose land would be expropriated is predicted to increase to 42 million by 2020, and the total number is expected to exceed 100 million by 2030 [1].

Landless peasants living in resettlement residential areas in China have been a long-standing problem. After the founding of the People’s Republic of China in 1949, a large scale of land has been expropriated for the construction of a new city. However, arable land was collectively operated, and problems about landless peasants had not been seriously considered. After the implementation of the Reform and Opening-up Policy in 1978, the Household Responsibility System was launched in rural China, which allowed peasants to regain their land rights. The rapid development of urbanization resulted in the separate occurrence of two waves of Rodeo Heat in the mid-1980s and the early 1990s. Landless peasants were rehoused under the government plans, and they were offered jobs by companies that expropriated their lands based on the hiring index. The third Rodeo Heat with a larger scale occurred in 2000, in which urban and industrial areas expanded at an unprecedented rate. Moreover, infrastructure construction required additional land. An increasing amount of collective land in rural China are being occupied and transformed into urban land using all types of methods, Traditional peasants used to make a living on land; however, an increasing number of peasants are losing their land [2].

Related with this issue is the expression and protection issue of landless peasants’ interests and demands. Influenced by the traditional ideology that Chinese people fear government officials and avoid political issues, the majority of landless peasants choose to remain silent about their interests. However, some individuals continue to choose to gather and initiate actions for their rights. For example, they express their problems to the upper government or turn to lawyers. The most extreme cases include certain individuals choosing to react uncompromisingly towards the situation by encouraging violent protests, demolition, some of which endanger the lives of many. These triggered aggressive events have evolved to be a major social concern. Examples are the reports of a female entrepreneur, Fuzhen Tang, in Chengdu City, Sichuan Province, who self-immolated in 2009; the self-immolation of three villagers in Yihuang County, Jiangxi Province in 2010; and in 2014 when a fire broke out in the tents of peasants in Pingdu City, Shandong Province. The major news regarding landless peasants protesting against demolitions have shocked not only the country, but the entire world, which has resulted in far-reaching impact. In recent years, similar extreme events can be often seen in newspapers, serving as a reminder of the concerns of landless peasants and the sense of urgency in the situation.

Studies on Chinese landless peasants can be classified into two categories. The first category mostly focuses on how and why peasants lost their land. For instance, The urban land area in the Beijing-Tianjin-Hebei region expanded by 71% between 1990 and 2000. Approximately 74% of the new urban land was converted from arable land, and smaller cities tended to have higher percentages. One of the important reasons for this result is that urban land is highly correlated with arable land in spatial distribution [3]. Some scholars investigated farmland acquisitions and urban land transactions in Yingtan City in Jiangxi Province. They revealed that the current policy of increasing urban land conveyance through competitive mechanisms cannot help better protect the landless peasants [4]. Deng et al. reported that income growth plays a powerful role in the urban expansion of China, followed by population, value of agricultural land, transportation costs, industrialization, and the rise of the service sector; the last two factors affect the growth of the urban core [5].

The second category of studies examines how landless peasants live. In places where dynamic industries are lacking and lineage/kinship organizations are weak, the outcomes of property rights changes incurred by land requisition are often negative and even lethal to the livelihood of landless peasants. This case is particularly true in the less developed areas in inland China, especially the western region, an area that remains comparatively under investigated and less familiar to non-Chinese specialists. The imperfect implementation of compensation institutions (and possibly their imperfect design) implies that sums do not stack up for many dispossessed urban villages. These villages become vulnerable to poverty because they privately bear a significant proportion of the transaction costs of urban expansion [6]. Social concerns are absorbed into the issues of social tension and justice [7], social exclusion [8], social security of landless peasants [9,10], and so on, all of which may pose a long-term threat to stability and sustainable development. Besides, urban integration and self-identity is one of the research directions of scholars, thought to be related to the temporality and spatiality transformation of landless peasants [11]. In general, the horizons of domestic research on the problem of landless peasants are relatively narrow because of the lack of comparative and systematic studies [2].

However, few studies focus on the health or quality of life (QOL) of Chinese landless peasants. QOL was developed by the World Health Organization (WHO). Certain scales have been widely used to measure the health-related quality of life (HRQOL) or QOL of China’s overall population. The Chinese version of European Quality of Life (EQ-5D) has been used in nationwide or cities’ investigations and has demonstrated acceptable construct validity and reliability [12-14]. The 36-item Short Form Health Survey (SF-36) is one of the popular scales. Many cities in China have applied the SF-36 scale. SF-12 is a short version of the SF-36 scale. The results can be considered reliable and sensitive in measuring the HRQOL of Chinese, while the SF-12 is an effective alternative of the SF-36.

The WHOQOL, an instrument for measuring QOL, assesses the perceptions of individuals regarding their position in life in the context of the culture and value systems in which they live and in relation to their goals, expectations, standards, and concerns. Because they refer to the international standards that are commonly used in the cross-cultural studies [15,16], the surveys help distinguish different vulnerable groups by epidemiological studies [17]. The WHOQOL Group first designed the WHOQOL-100 scale with 100 items, which consisted of 24 facets. Each facet has four questions. Moreover, four questions cover general QOL and health. The scale was aimed to measure people’s QOL comprehensively. However, the WHOQOL-100 is lengthy and not suitable for the practical application. On the basic of WHOQOL-100 scale, WHOQOL-BREF is developed [18], which is composed of 26 items. Among them, two items measure the evaluation of general QOL and health. The 24 items can be divided into four domains. The measurement scores of two scales are substantially similar [19]. Therefore, the WHOQOL-BREF is a reliable and effective alternative of the WHOQOL-100 scale [20]. In consideration of reducing research costs or event rates that the hesitancy of respondents to finish the questionnaire because of the length, the majority of researchers choose the shorter version, the WHOQOL-BREF scale.

In 1996, the WHOQOL-BREF scale was available in 19 different languages. Several countries proved the scale to be reliable and valid [21-25]. In 2004, the WHOQOL-BREF was available in 40 countries, translated in most languages [19]. One cross-cultural investigation indicated that WHOQOL-BREF was a sound and valid assessment of QOL [26]. The WHOQOL-100 and other shorter versions of the survey, particularly in the Chinese mainland, were translated by a research group from the School of Public Health, Sun Yat-sen University in 2000 [27]. They are also used in extensively measuring the QOL among Chinese, such as urban community residents [28], depressed older people [29], rural–to–urban migrants [30,31], patients with chronic diseases and their caregivers [32], and rural community residents after an earthquake [33].

Other methods are likewise used to measure the QOL of Chinese peasants; however, these methods mostly focus on specific groups, such as children living in HIV/AIDS-affected families [34] and older populations [35]. Only a few studies focus on the QOL of Chinese landless peasants. The current study attempts to fill the gap in the literature using the WHOQOL-BREF to measure the QOL of Chinese landless peasants, and conducts a comparison between male and female peasants to determine health inequity among Chinese landless peasants.

As characteristics of a large vulnerable group, women’s physical health is widely acknowledged to be in an unfavorable state. The reasons involved are diverse and complex, including social, political, cultural, and personal factors [36]. They can be divided into four facets: discriminatory values, norms within households and communities, differential exposures and vulnerability to diseases, biases in health system and biased health research [37]. Although feminism has progressed with advances in civilization and technology, some aspects have changed significantly. Reevaluating this view becomes necessary [38]. Still many studies that focused on the health of specific populations found that women’s health was in disadvantaged conditions; the objects include earthquake survivors [39,40], different groups of patients [41,42], and general population with different ages [43,44]. Gender inequity not only hurts the health of millions of women, but also brings indirect disadvantages to men, such as more risky and unhealthy behaviors [37]. For Chinese women living in rural areas, this situation is even more complicated. The rapid economic development with the country’s participation in the globalization process has brought a double-edged effect on rural women’s health. On one hand, eliminating absolute poverty helps reduce the prevalence rate of rural women; on the other hand, young men have left their homes to work in factories in cities, leaving their families behind. Many women had to shoulder several responsibilities such as attending to families, farming, and household chores. Even for women who left their homes to work, they are forced to overwork in order to earn a living. Therefore, in either circumstance, rural women are faced with severe physical, psychological, and social health crises [45]. As a result, combining the above-mentioned concerns regarding the health of peasants, we considered the health of female landless peasants to be worthy of our study.

Early studies have indicated that China achieved significant progress in women’s health in the 1950 to 1990 period; nevertheless, far less was achieved with respect to gender equality overall, and a disparity in health between men and women remained [46]. A survey conducted in the late 1990s in Hebei Province likewise cited gender differences in health, and age was related to the size of gender difference [47]. A study in 2008 reported that women were less likely to learn information about tuberculosis and share it with others on their own initiative, and a larger proportion of women preferred to initially visit the lower-level non-hospital health facilities, such as village clinics and drugstores [48]. QOL is closely related to health. Thus, we propose the first hypothesis in this study.

***Hypothesis 1****: Chinese landless female peasants have a lower QOL than their male counterparts.*

In rural China, several social class indicators (i.e., education, income, and occupation) are associated with health [49]. In rural China, girls experience more difficulty in obtaining education compared to boys [50-53]. Moreover, education-related gender differences in health exist [54]. And eduction is a powerful indicator for health inequality in rural China [55]. Based on the foregoing analysis, we propose the second hypothesis in this study.

***Hypothesis 2****: Different domains of QOL demonstrate gender disparity among Chinese landless peasants.*

Peasants’ loss of land is an inevitable phenomenon in the development of society and urbanization. Peasants not only lose land and natural resources that they use to make a living, but also face an awkward situation in which they have no rights to share the achievements of urbanization because of institutional constraints. Finally, they are abandoned by urbanization and lose any livelihood security. Determining their QOL should be of social concern. This study uses the WHOQOL-BREF scale to measure the QOL of Chinese landless peasants, and analyzes gender differences. It aims to (1) assess the QOL of Chinese landless peasants and raise the social concern for these peasants; (2) explore health inequity among genders and promote special concern for Chinese landless female peasants; and (3) provide policy suggestions for government to improve health equality among landless rural peasants.

## Methods

### Samples and sampling procedure

The Yangtze River Delta (YRD) region is located in the fan-shaped alluvial plain where the Yangtze River runs into the East China Sea. The YRD region comprises 30 cities led by Shanghai, and has acreage of 210,000 square meters and a population of 159 million. This region absorbs several types of industries, economies, trades, education, technologies, culture, and so on. It has been engineered to pull the economy of the Yangtze River basin, connecting domestic with foreign market, absorbing foreign investment, promoting industrial and technological transfer, and joining international competition and regional reconstruction. Rapid economic development is inevitably accompanied by the rapid expansion of urbanization.

**Figure 1:**
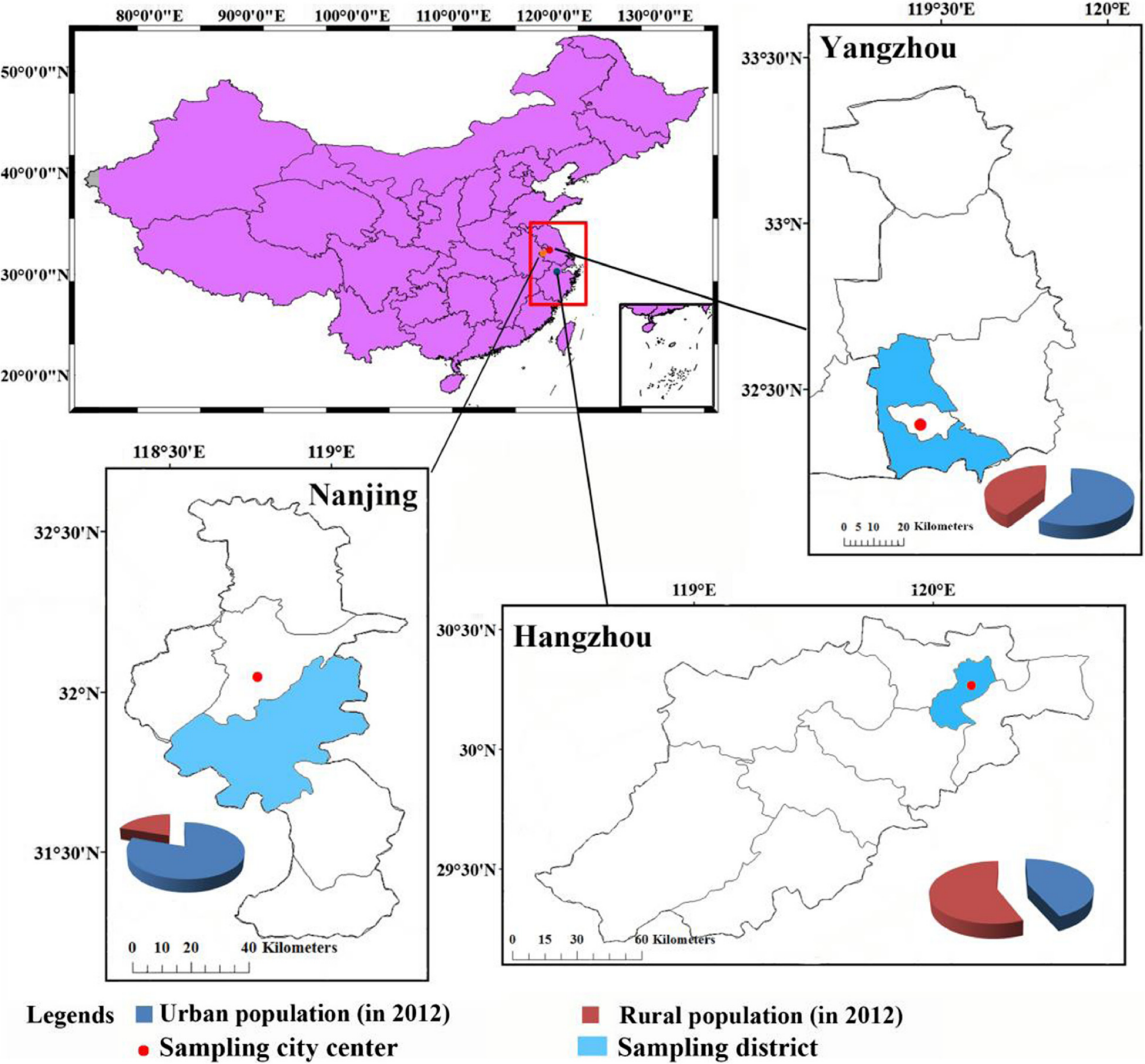
Geographical location and population of three sampling cities.

***First***, we used a non-random (non-probability) sampling method in selecting three cities (Yangzhou City in Jiangsu Province, Nanjing City in Jiangsu Province, and Hangzhou City in Zhejiang Province) in 30 cities of the YRD region (Figure 1). These three cities were selected because they had a GDP growth rate of approximately 10% in 2012, indicating their rapid development [56]. According to the 2012 annual data of local Bureaus of Statistics, the proportions of urban population of Nanjing and Yangzhou cities are higher than that those of rural population. And Hangzhou City has more rural populations. Samples taken in these three cities not only contribute a high quality of representativeness, but also produce certain reference values on how to improve the QOL of landless peasants with a larger scale in China.

***Second***, according to the information provided by the local Ministry of Civil Affairs, landless peasants can obtain a certain amount of money as compensation for the loss of their land; meanwhile, arrangements to move to a community are made for the landless peasants. We used a random (probability) sampling method in selecting the resettlement residential areas; two were chosen from all communities that are built to settle landless peasants in each city (two communities in Hanjiang District, Yangzhou City; two communities in Jiangning District, Nanjing City; two communities in Xihu District, Hangzhou City), as illustrated in Figure 1.

Interviewers with professional training skills administered household surveys to landless peasants living in these communities. Households were stratified by the number of adults in the household, and a Kish grid sampling method was used to perform equal probability sampling within each stratum. The results of a health survey in China indicated that the demographic characteristics of age and gender of the Kish grid sample approximated to the population, suggesting that the Kish grid is a sampling method that is practicable and easy to monitor. Moreover, this method can effectively decrease the bias derived from the sampling procedure within households [57]. Each questionnaire was independently completed by a respondent under the instructions of the interviewer. Up to 1,500 questionnaires were distributed, or 500 questionnaires in each city. We collected 1,236 valid questionnaires, and derived an effective collection rate of 82.4%.

The current study principally measured the QOL of landless peasants in the YRD region using the WHOQOL-BREF scale. The WHOQOL-BREF contains four domains, namely, physical health, psychological health, social relationships and environment. These domains have 26 items. Three of these items are reverse–scored and two are overall questions that yield information on global QOL and health. The reverse items are converted according to the method shown in the WHOQOL instructions. The four domains have the following number of items: seven (Q3, Q4, Q10, Q15, Q16, Q17, Q18), six (Q5, Q6, Q7, Q11, Q19, Q26), three (Q20, Q21, Q22), and eight (Q8, Q9, Q12, Q13, Q14, Q23, Q24, Q25), respectively. We normalize the obtained data (Formula 1) to facilitate the comparison of all domains. Final scores are in the range of 0 and 100. Higher scores indicate that the respondents have a higher QOL.

Formula 1:

$$\mathrm{Converted}\;\mathrm{scores}=\left(\mathrm{Raw}\;\mathrm{scores}-4\right)*\left(100/16\right)$$

As compared with the SF-36 scale, another international popular scale with strong reliability and validity, the WHOQOL-BREF has fewer ceiling effects [58]. The SF-36 measures the HRQOL instead of the global QOL [59]. Table 1 shows a detailed comparison of Spearman correlation coefficients between two scales. In general, the correlation coefficients of each domain of two scales are relatively high. For example, the correlation coefficients between four domains of WHOQOL-BREF and physical functioning (PF) are all above 0.730, indicating high correlations. The correlations between four domains of WHOQOL-BREF and role physical (RP) are strong; their coefficients are all above 0.640. Generally, if the domains of two scales have overlapping portions, the correlations are strong; if the domain of one scale is not mentioned in another scale, the correlation is weak. For example, the correlation coefficient of each domain of WHOQOL-BREF and Vitality (VT) of SF-36 scale are approximately 0.1, which is relatively low. In summary, the analysis results of scales indicate strong reference values.

**Table 1:** Comparison of the correlations coefficients between two scales

Physical health	0.784	0.694	0.658	0.195	0.109	0.268	0.667	0.402
Social relationships	0.790	0.680	0.648	0.204	0.088	0.282	0.674	0.390
Social relationships	0.738	0.643	0.615	0.219	0.100	0.251	0.645	0.356
Environment	0.797	0.679	0.653	0.194	0.107	0.269	0.689	0.405
	PF	RP	BP	GH	VT	SF	RE	MH

Note: In the eight domains of SF-36 scale, PF: Physical functioning; RP: Role physical; BP: Bodily pain; GH: General health; VT: Vitality; SF: Social functioning; RE: Role emotional; MH: Mental health.

**Figure 2:**
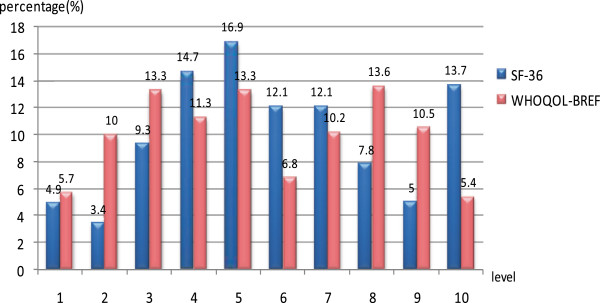
Comparison of SF-36 and WHOQOL-BREF scores.

Figure 2 illustrates the difference of proportion of score between SF-36 and WHOQOL-BREF. The ranges of transferred scores of two scales are 0 to 100. Recoding the scores can be transferred into ten levels from 1 to 10 as illustrated in the horizontal axis and the vertical axis indicates the population percentage in this score. The higher the bar graph is, the more people scored HRQOL in this level. As illustrated in Figure 2, the number of people who scored in the highest or lowest level is minimal, compared to the people who scored in the middle level. Comparison indicates subtle differences in the levels between two scales. One example is the number of people who scored the fifth level in SF-36 scale peaks, whereas that of WHOQOL-BREF does not have any apparent peaks. The bars in No. 3, 5, 8 of WHOQOL-BREF scores are balanced, indicating high proportions.

### Analysis methods

#### Reliability analysis

The reliability and validity of questionnaires are tested after their completion. Reliability test has nothing to do with the correctness of the measurement results obtained; instead, it examines whether or not the measurement itself is stable. The most commonly used method is Cronbach’s α coefficient, which principally tests the internal reliability of the scale and determines any internal consistency among items in the scale. This coefficient is always believed to be between 0 and 1. A value larger than 0.9 indicates that the reliability test result is very good; a value between 0.8 and 0.9 indicates that the reliability test result is acceptable; a value between 0.7 and 0.8 indicates that some items in the scale need to be revised; and a value less than 0.7 indicates that some items in the scale need to be moved. Reliability analysis can help judge the stability and reliability of a scale.

#### Analysis of variance

Analysis of variance (ANOVA) is a method to determine whether the difference of a mean value of two or more of generals have statistical significances. To increase the effectiveness of ANOVA, we hypothesized that the compared generals have the same variances. The difference of health between male and female landless peasants will be studied. Thus, the homogeneity of variance tests will be conducted first, then the ANOVA.

#### Nonparametric tests

ANOVA needs to make hypotheses on the nature of general samples; that is, to introduce certain limitations to the distribution shape of generals. When the variances of one domain of male and female generals are not equal, ANOVA is therefore not appropriate. The non-parametric tests do not involve the general parameters. As such, the test conditions are lenient and adaptable. Moreover, the method is more flexible and versatile. The specific non-parametric test used in this study is the Mann–Whitney U test. The test is conducted by studying the average rank of two groups of samples. The original hypothesis is that no significant differences occur on the distribution of generals between two groups of independent samples.

#### Confirmatory factor analysis

A confirmatory factor analysis (CFA) model determines the true population variance, covariance, estimated population variance, sample covariance, and estimated covariance, as well as the related overall difference and the approximated, estimated, and sample differences. Goodness of fit is an indicator for testing the similarity between the model-estimated covariance matrix and sample covariance. Chi square to degree of freedom ratio (χ^2^/df) is an indicator for testing the similarity between the sample covariance matrix and the estimated covariance matrix, and its theoretical expected value is 1. If χ^2^/df is closer to 1, then the sample covariance matrix and estimated covariance matrix are more similar. In a practical study, when χ^2^/df is closer to 2, the model demonstrates better fit. When the scale of a sample is large, then the value of χ^2^/df approximately 5 is acceptable.

#### Gender differences analysis based on CFA

Confirmatory factor analysis (CFA) is a special application of structural equation modeling (SEM) analysis. It is a factors analysis procedure that explores whether the factor structure of the scale model is collected fit the actual data, whether the index of scalar can be effectively used as a factor dimensions (latent variable) measuring variables. In this study, we use second-order confirmatory factor analysis in structural equation modeling to test the appropriateness and authenticity of the established factors structural models. Besides, we explore the path coefficients of female and male’s various domains which affect the quality of life. Through the processing of the WHOQOL-BREF scale data, we calculate male and female’s composite scores in four domains, build mutual relations in various domains. Thus, form the corresponding theoretical assumptions, and the path coefficients to estimate the theoretical model.

### Ethical statement

Research involving human subjects (including human material or human data) in this study has been performed with the approval of ethnics committee of the School of Social and Behavioral Sciences, Nanjing University. Research carries out on humans has been in compliance with the Helsinki Declaration. And the authors would take the interpretation and responsibility for results involving human subjects in this study.

### Consent statement

Before respondents filled in the questionnaire in this study, they had been told that their data would be used only for academic research, and they ensured that their information filled in the questionnaire was in accordance with the actual situation. Where participants are children, their parent or guardian was consent for the participation.

## Results

### Descriptive statistics

Table 2 shows the results of the comparative analysis of the descriptive statistics in the four domains of the WHOQOL-BREF scale (N=1236). Specifically shown from top to bottom are the mean values, median values, plural values, variance, skewness, kurtosis, full distance, minimum values, and maximum values.

**Table 2:** Descriptive statistics of the WHOQOL-BREF scale

		**Physical health**	**Psychological health**	**Social relationships**	**Environment**
N	Valid	1236	1236	1236	1236
	Missing	0	0	0	0
Mean value		46.3169	47.2594	50.7723	47.6517
Median value		46.4300	41.6700	50.0000	43.7500
Plural value		32.14	29.17	83.33	78.13
Variance		591.825	681.694	807.939	639.800
Skewness		.091	.258	-.033	.109
Standard error of skewness		.070	.070	.070	.070
Kurtosis		−1.129	−1.252	−1.238	−1.242
Standard error of kurtosis		.139	.139	.139	.139
Full distance		100.00	95.83	100.00	96.88
Minimum value		.00	4.17	.00	.00
Maximum value		100.00	100.00	100.00	96.88

The results indicated unsatisfactory scores in the four domains of WHOQOL-BREF among landless peasants. Under the 100-score scale, the highest mean value was 50.7723 in the domain of social relationships, and the other three domains all obtained scores lower than 50. Four domains exhibited a impact on the QOL of landless peasants.

In the plural value statistics, most of the respondents obtained scores of 32.14 in physical health and 29.17 in psychological health; meanwhile, scores in social relationships and environment were higher than the former two. In the variance statistics, the score in social relationships was the highest at 807.939, which indicated a difference with the other three domains. In other words, data in these three domains were more stable than those in social relationships. In the kurtosis statistics, scores in the four domains were all less than 0, which indicated that data distribution was not as steep as the standard normal distribution, but was slightly smooth. Moreover, no great differences were found in the full distance, minimum value, and maximum value statistics among the four domains.

Comparison of data showed that landless peasants obtained the lowest mean scores in physical health (46.3169), and a median value of 46.4300, with a slight difference with the mean value. The value of variance in physical health was the lowest among four domains, indicating that landless peasants obtained a relatively concentrated score of 46, with less volatile data and a smaller difference in scores. In social relationships, the mean value was 50.7723, median value was 50.0000, and variance obtained the largest value, indicating that data in this domain exhibited a difference. In other words, landless peasants demonstrated the lowest consistency when assessing their attitude in this domain.

### Homogeneity of variances

As can be seen in Table 3, only the *P*-values of probability, which are above 0.05, correspond to physical health and social relationships. Thus, the variances of physical health and social relationships under different genders are the same and will satisfy the prerequisites of ANOVA. The *P*-value of another two variables show to be less than the significance level, which means the variances of different genders are not the same. Accordingly, the ANOVA cannot be adopted. We used One-way ANOVA to analyze whether gender has significant differences in terms of physical health and social relationships, and used Mann-Whitney U nonparametric test to study whether gender has significant differences with the remaining variables Table 4 shows the results of physical health and social relationships for ANOVA.

**Table 3:** Test results for homogeneity of variances

	**Levene statistic**	**df1**	**df2**	**Sig.**
Physical health	1.577	1	1111	.209
Psychological health	34.126	1	1073	.000
Social relationships	1.076	1	1148	.300
Environment	6.991	1	1084	.008

**Table 4:** Results for ANOVA

		**Sum of squares**	**df**	**Mean square**	***F***	**Sig.**
Physical health	Between Groups	209854.972	1	209854.972	568.118	.000
	Within Groups	410388.395	1111	209854.972		
	Total	620243.367	1112			
Social relationships	Between Groups	244473.315	1	244473.315	398.830	.000
	Within Groups	703696.975	1148	612.976		
	Total	948170.290	1149			

The sum of squared variations of physical health is 620243.367. If only one factor (gender) is considered, among the total variation of physical health, the interpretable variation of different gender is 209854.972; the variation caused by sampling errors is 410388.395. Their variances are 209854.972 and 209854.972, respectively. The *F*-statistic result acquired by division is 568.118. The corresponding P-value is approximately 0. If the significance level (α) is 0.05, because the *P*-value is less than α, the original hypothesis is rejected. Hence, significant differences occur between the impacts of different genders on physical health.

The sum of squared variation of social relationships is 948170.290. If only one factor (gender) is considered, among the total variation of social relationships, the interpretable variation of different gender is 244473.315; the variation caused by sampling errors is 703696.975. Their variances are 244473.315 and 612.976, respectively. The *F*-statistic result acquired through the division is 398.830. The corresponding P-value is approximately 0. Because the *P*-value is less than α, the original hypothesis is rejected. Hence, significant differences occur between the impacts of different genders on social relationships.

### Nonparametric tests

As demonstrated by the Mann-Whitney U nonparametric test result in Table 5, the differences of the psychological health and environment under different genders are significant. Combining the results of ANOVA in Table 4, the differences of the four domains of WHOQOL-BREF under different genders are significant.

**Table 5:** Result of Mann-Whitney U nonparametric tests

	**Psychological health**	**Environment**
Mann–Whitney U	49166.500	52655.500
Wilcoxon W	170937.500	168576.500
Z	−18.615	−18.098
Asym.Sig	.000	.000

### Reliability analysis

In Table 6, the Cronbach’s α coefficients (third column) in the four domains of the WHOQOL-BREF scale are considerably larger than 0.800, indicating that each item in the scale shows high internal consistency and good reliability. Meanwhile, the overall Cronbach’s α coefficient is 0.926, and becomes 0.928 after being standardized. Reliability coefficient is larger than 0.8, indicating good internal reliability and high internal consistency of the scale. In other words, this scale has high value.

**Table 6:** Reliability test

**Domains**	**Number of observed variables**	**Cronbach’s α coefficient if the items are removed**
Physical health	7	0.909
Psychological health	6	0.893
Social relationships	3	0.922
Environment	8	0.892

Moreover, if the items are removed, the Cronbach’s α coefficient of each domain is less than the overall one, 0.926. When the physical health, psychological health, social relationships, and environment are separately removed, the Cronbach’s α coefficients are 0.909, 0.893, 0.922, and 0.892, respectively. If the items are removed, all Cronbach’s α coefficients are less than the overall coefficient of 0.926, which indicates that the separate removal of any domain is meaningless. These four domains are closely connected with each other.

Scores in the WHOQOL-BREF scale are positive values; a higher score indicates a better QOL. Table 7 shows the gender difference in the scores of four domains of Chinese landless peasants. Under the 100-score scale, landless peasants commonly obtain low scores in each QOL domain, whereas males obtain higher scores than females. Compared with males, females obtain lower scores of 27.64 in psychological health, 30.51 in physical health, 28.84 in social relationships, and 28.69 in environment. Males obtain much higher scores of nearly 30 than females in each domain, indicating a great gender difference in the QOL.

**Table 7:** Gender difference in the QOL of Chinese landless peasants

**Domains**	**Mean value (male)**	**Mean value (female)**	**Variance (male)**	**Variance (female)**
Psychological health	59.90	32.26	20.96	19.05
Physical health	62.18	31.67	23.36	18.76
Social relationships	64.97	36.13	25.39	23.49
Environment	61.71	33.02	21.98	19.40

When all landless peasants demonstrate low scores in the QOL, males obtain higher scores than females, indicating a considerable gender difference in each QOL domain. Females obtain lower scores of variance in the four domains than males, indicating a lower difference in the scores of four domains. In others words, the degree of concentrated scores in each domain is higher among females than males. Meanwhile, females obtain lower mean values than males, indicating that females obtain lower scores in each domain. Thus, gender difference affects the QOL among Chinese landless peasants.

**Figure 3:**
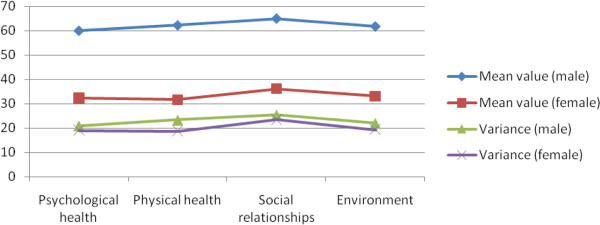
Comparison of the four domains of QOL under gender differences.

As indicated in Figure 3, the mean value is small, indicating that landless female peasants are disadvantaged in the four domains of QOL. And the variance of males is greater than that of females, indicating that the fluctuation of men's scores are greater than that of women's scores.

### CFA model

**Figure 4:**
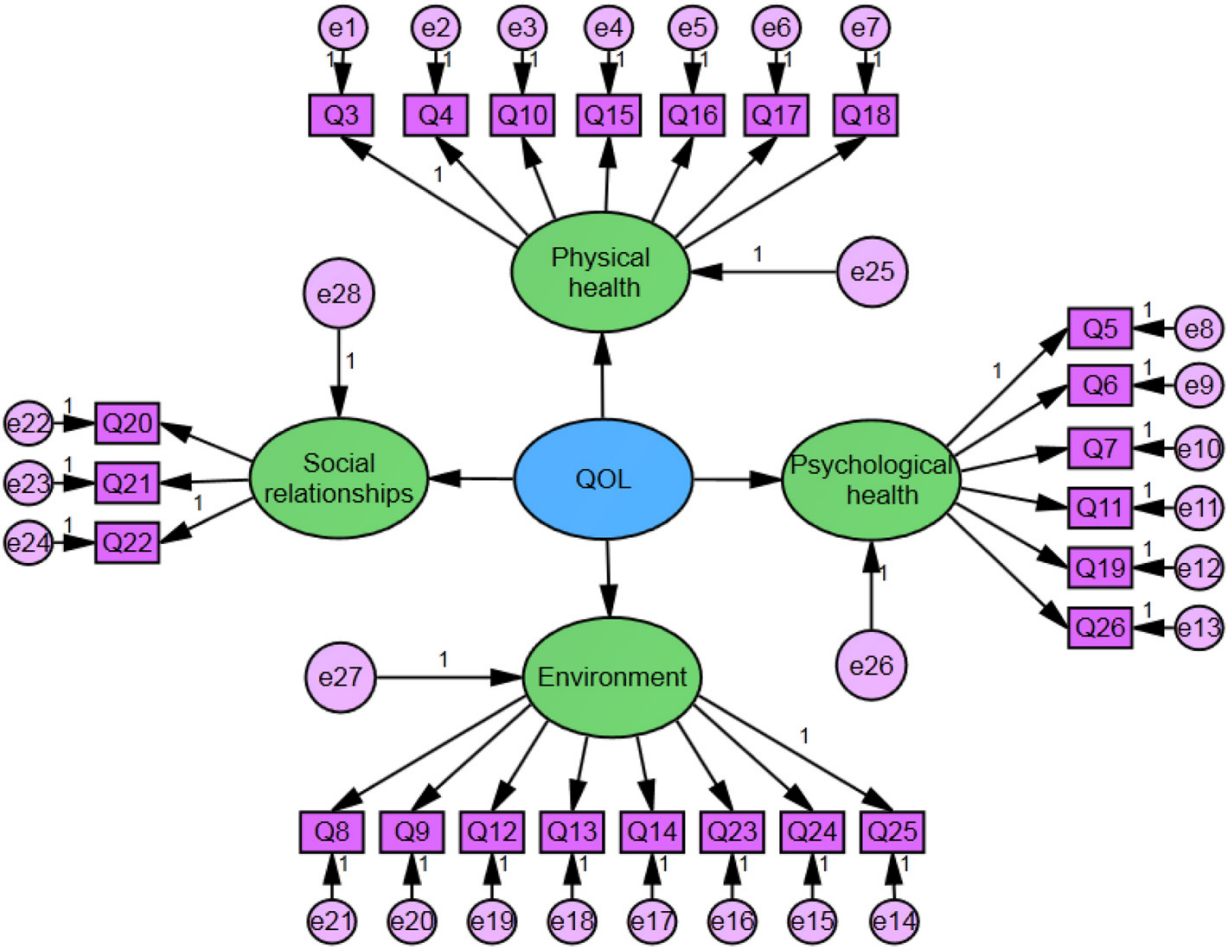
**CFA model of the WHOQOL-BREF scale for the female landless peasants.** Note: Q3-Q26Indicate the 24 items of WHQOL-BREF scale. Q3: Pain and discomfort; Q4: Dependence on medicinal substances and medical aids; Q5: Positive feelings; Q6: Spirituality / Religion / Personal beliefs; Q7: Thinking, learning, memory and concentration; Q8: Freedom, physical safety and security; Q9: Physical environment (pollution / noise / traffic / climate); Q10: Energy and fatigue; Q11: Bodily image and appearance; Q12: Financial resources; Q13: Opportunities for acquiring new information and skills; Q14: Participation in and opportunities for recreation / leisure activities; Q15: Mobility; Q16: Sleep and rest; Q17: Activities of daily living; Q18: Work Capacity; Q19: Self-esteem; Q20: Personal relationships; Q21: Sexual activity; Q22: Social support; Q23: Home environment; Q24: Health and social care: accessibility and quality; Q25: Transport; Q26: Negative feelings.

Figure 4 shows the CFA model for the WHOQOL-BREF scale. In this model, the QOL is a latent variable, and its indicators consist of four domains that are connected by a single-direction arrow. Moreover, the fitting indices for comparing the CFA model with the reference standard are the root mean residual (RMR), the root mean square error of approximation (RMSEA), goodness of fit index (GFI), adjusted goodness of fit Index (AGFI), normed fit Index (NFI), incremental fit index (IFI), Tucker-Lewis Index((TLI) non-normed fit Index (NNFI), comparative fit index (CFI), parsimonious GFI (PGFI), parsimonious NFI (PNFI), parsimonious CFI (PCFI).

Table 8 shows the CFA analysis results of the WHOQOL-BREF scale, including parameter estimation, CR coefficient, accompanied probability between four domains and QOL, as well as between certain items and their related domains. According to the results of the former four columns, the parameter estimation values are all larger than 1, indicating that the related domains can reflect the QOL of the measure group. The CR coefficients are all larger than 2, indicating that most accompanied probabilities passed the significance tests, and they demonstrate good analysis results. Factor analysis results between certain items and their related domains exhibit a relatively large value of parameter estimation (only one is less than 0.80, and others are larger than 0.80). Most CR coefficients are larger than 18, indicating the high validity of the scale. Analysis results indicate significant implications for the QOL among landless female peasants in living in the resettlement residential areas in China.

**Table 8:** CFA analysis of the WHOQOL-BREF scale

			**Estimate**	**S.E.**	**C.R.**	**P**
Physical health	<−−-	QOL	1.000			
Social relationships	<−−-	QOL	1.144	.058	19.660	***
Psychological health	<−−-	QOL	1.154	.057	20.108	***
Environment	<−−-	QOL	1.120	.058	19.416	***
Q16	<−−-	Physical health	1.000			
Q15	<−−-	Physical health	1.028	.057	18.087	***
Q10	<−−-	Physical health	1.026	.057	18.001	***
Q4	<−−-	Physical health	.823	.055	15.038	***
Q3	<−−-	Physical health	1.042	.057	18.324	***
Q18	<−−-	Physical health	1.142	.058	19.632	***
Q17	<−−-	Physical health	.981	.057	17.121	***
Q22	<−−-	Social relationships	1.000			
Q21	<−−-	Social relationships	.911	.041	22.174	***
Q20	<−−-	Social relationships	1.080	.044	24.763	***
Q5	<−−-	Psychological health	1.000			
Q6	<−−-	Psychological health	.844	.044	19.352	***
Q7	<−−-	Psychological health	1.018	.044	22.888	***
Q11	<−−-	Psychological health	.747	.040	18.514	***
Q19	<−−-	Psychological health	.909	.044	20.868	***
Q26	<−−-	Psychological health	.980	.041	23.978	***
Q8	<−−-	Environment	1.000			
Q9	<−−-	Environment	.934	.047	19.925	***
Q12	<−−-	Environment	.944	.048	19.701	***
Q13	<−−-	Environment	.938	.049	19.239	***
Q14	<−−-	Environment	.833	.046	18.275	***
Q23	<−−-	Environment	1.118	.048	23.448	***
Q24	<−−-	Environment	1.088	.049	22.332	***
Q25	<−−-	Environment	.899	.044	20.459	***

Note: ***indicates the path coefficients are significant when their confidence interval is over 99%. Q3-Q26Indicate the 24 items of WHQOL-BREF scale. Q3: Pain and discomfort; Q4: Dependence on medicinal substances and medical aids; Q5: Positive feelings; Q6: Spirituality / Religion / Personal beliefs; Q7: Thinking, learning, memory and concentration; Q8: Freedom, physical safety and security; Q9: Physical environment (pollution / noise / traffic / climate); Q10: Energy and fatigue; Q11: Bodily image and appearance; Q12: Financial resources; Q13: Opportunities for acquiring new information and skills; Q14: Participation in and opportunities for recreation / leisure activities; Q15: Mobility; Q16: Sleep and rest; Q17: Activities of daily living; Q18: Work Capacity; Q19: Self-esteem; Q20: Personal relationships; Q21: Sexual activity; Q22: Social support; Q23: Home environment; Q24: Health and social care: accessibility and quality; Q25: Transport; Q26: Negative feelings.

***First***, psychological health shows the greatest impact on the QOL of landless peasants, with a coefficient of 1.154. The lack of concern for psychological health has negatively affected the QOL of landless peasants. In rural China, peasants care little about their psychological health and typically ignore problems related to their psychological wellbeing. These behaviors decrease their QOL. Thus, examining the QOL among landless female peasants is practical and valuable.

***Second***, the domain of social relationships shows the second greatest impact on the QOL of landless peasants, with a coefficient of 1.144. This result is understandable. When their land is expropriated, peasants lose their means of livelihood and experience changes in their social relationships, thereby significantly affecting their QOL. If their social relationships are unsatisfactory and the necessary social support is missing, their QOL will decrease. Thus, a discussion on solving strategies should raise awareness of the contribution of the loss of land to the lack of a social support system. Women are more likely to have weak social relationships because of their more disadvantaged social status. The loss of land connotes the loss not only of a source of livelihood, but also of normal social relationships. The domain of social relationships should be considered in undertaking efforts to improve the QOL of landless female peasants.

***Third***, physical health shows a relatively weak impact on the QOL of landless peasants. Physical pain and exhaustion may not be the principal QOL factors, and physical health does not directly affect their lives. Moreover, physical health may show a long-term slow impact on QOL. Thus, its parameter estimation value is relatively lower than the other domains.

***Four***, the domain of environment shows the third greatest impact on the QOL of landless peasants, with a coefficient of 1.120. This result is reasonable. When they become landless peasants, their living environments change, both social and natural, hence significantly affects their QOL.

Table 9 shows the fitting indices to the second-order CFA model of the WHOQOL-BREF scale. The first column shows the fitting indices, the second column shows the reference standards for the model, the third column shows the testing results of the model, and the fourth column shows whether or not the testing results for the model are appropriate. In Table 9, most fitting indices to the model reach an acceptable standard, indicating that the model fits well, and its external quality and convergent validity are good. Meanwhile, χ^2^ is 748.078, df is 248, and χ^2^/df is 3.016, indicating that the overall model fits well and is acceptable.

**Table 9:** Fitting indices to the second-order CFA of the WHOQOL-BREF scale

**Fitting indices**	**Testing results**	**Testing results**	**Fitness**
***χ***^***2***^		748.078	Yes
***χ***^***2***^ ***/df***		3.016	Yes
**RMR**	<0.05	0.0301	Yes
**RMSEA**	0.08 (<0.05: excellent; <0.08: good)	0.040	Yes
**GFI**	>0.90	0.944	Yes
**AGFI**	>0.90	0.932	Yes
**NFI**	>0.90	0.940	Yes
**IFI**	>0.90	0.959	Yes
**TLI(NNFI)**	>0.90	0.955	Yes
**CFI**	>0.90	0.959	Yes
**PGFI**	>0.50	0.780	Yes
**PNFI**	>0.50	0.845	Yes
**PCFI**	>0.50	0.862	Yes

#### Gender differences analysis based on CFA

Confirmatory factor analysis (CFA) is actually a special application of structure equation modeling (SEM) analysis. On the analysis of the above, second-order confirmatory factor analysis is used in structural equation modeling analyzing the appropriateness and authenticity of the established factors structural models. What’s more, based on the above analysis, we explore the path coefficients of female and male’s various domains which affect the quality of life. For each observed variable, data are distributed with normality, which meet the requirements of the model. A review of the previous literature and preprocessing of WHOQOL-BREF data allow the composite scores of four domains to be calculated, the correlations among domains to be established, the theoretical hypotheses to be tested, and the path coefficients of the theoretical model to be estimated. The estimated results are shown as follows.

(1) $$\begin{array}{ll} \mathrm{QOL}\;\left(\mathrm{male}\right) & =1.000\times \mathrm{Physical}\;\mathrm{health}+1.254\\ & \;\times \mathrm{Psychological}\;\mathrm{health}+1.230\\ & \;\times \mathrm{Social}\;\mathrm{relationships}+1.205\\ & \;\times \mathrm{Environment} \end{array}$$

From Equation 1, when other domains remain unchanged, scores in physical health increase with 1, and the QOL of males increases with 1; when scores in psychological health increase with 1, the QOL of males increases with 1.254; scores in the domain of social relationships increase with 1, the QOL of males increases with 1.230; and when scores in the environment domain increase with 1, the QOL of males increases with 1.205. Psychological health shows the greatest impact on the QOL of landless male peasants, followed by social relationships, environment, and physical health.

(2) $$\begin{array}{ll} \mathrm{QOL}\;\left(\mathrm{female}\right) & =1.000\times \mathrm{Physical}\;\mathrm{health}\\ & \;+1.177\times \mathrm{Psychological}\;\mathrm{health}\\ & \;+1.098\times \mathrm{Social}\;\mathrm{relationships}\\ & \;+1.057\times \mathrm{Environment} \end{array}$$

Similar to Equation 1, in analyzing Equation 2, under the situation that other domains remain unchanged, scores in physical health increase with 1, and the QOL of females increases with 1; when scores in psychological health increase with 1, the QOL of females increases with 1.177; scores in the domain of social relationships increase with 1, the QOL of females increases with 1.098; and when scores in the environment domain increase with 1, the QOL of females increases with 1.057. Psychological health shows the greatest impact on the QOL of landless female peasants, followed by social relationships, environment, and physical health.

In the comparison of data from Tables 10 and 11, each domain shows a positive correlation with the QOL of landless male and female peasants. However, when scores in each domain increase with 1, the QOL increases more substantially among males than females. In other words, when psychological health, social relationships, environment, and physical health improve, the QOL of males improves more considerably than that of females. If females desire a similar improvement in their QOL, they have to make more positive changes in psychological health, social relationships, environment, and physical health. They will experience more difficulties in attempting to improve their QOL. Thus, the QOL of females should be given more attention.

**Table 10:** Path coefficients of each domain on the QOL of landless male peasants

	**Estimate**	**S.E.**	**C.R.**	***P***
Physical health < −−-QOL	1.000			
Psychological health < −−-QOL	1.254	.102	12.037	***
Social relationships < −−-QOL	1.230	.100	12.600	***
Environment < −−-QOL	1.205	.101	11.972	***

Note: ***indicates the path coefficients are significant when their confidence interval is over 99%.

**Table 11:** Path coefficients of each domain on the QOL of landless female peasants

	**Estimate**	**S.E.**	**C.R.**	***P***
Physical health < −−-QOL	1.000			
Psychological health < −−-QOL	1.177	.150	7.870	***
Social relationships < −−-QOL	1.098	.141	7.766	***
Environment < −−-QOL	1.057	.142	7.463	***

Note: ***indicates the path coefficients are significant when the confidence interval is over 99%.

The key findings in Table 12 are as follows.

**Table 12:** Proportions of path coefficients in gender difference

	**Physical health**	**Psychological health**	**Social relationships**	**Environment**
QOL (male)	21.33%	26.74%	26.23%	25.70%
QOL (female)	23.08%	27.17%	25.35%	24.40%

***First***, differences in paths coefficients are shown on the QOL of both males and females. For males, psychological health shows the greatest impact on the QOL, with a proportion of path coefficients of 26.74%, followed by social relationships (26.23%) and environment (25.70%); physical health shows the least impact with a proportion of 21.33%. For females, psychological health shows the greatest impact on QOL, with a proportion of path coefficients of 27.17%, followed by social relationships (25.35%) and environment (24.40%); physical health shows the least impact with a proportion of 23.08%.

***Second*,** the importance of the four domains affecting QOL is the same for landless male and female peasants. The proportion of path coefficients signifies that psychological health shows the greatest impact on the QOL of males (26.74%) and females (27.17%). The domain of social relationships shows the second greatest impact on the QOL of males (26.23%) and females (25.35%), followed by environment (25.70% for males, 24.4% for females) and physical health (21.33% for males, 23.08% for females).

***Third***, the proportions of the four domains are different. The gap between the maximum and minimum proportions is 5.41% for males and 4.09% for females. Thus, the four domains demonstrate an average impact on the QOL of females, indicating their importance.

## Discussion

The rapid development of urbanization in China has caused a larger number of peasants to lose their lands, with no rights to share the achievements of urbanization because of institutional constraints. In the process of land acquisition with great interests, these peasants are often in a passive and weak position, losing land to protect future life. This study assesses the QOL of landless peasants in the YRD region, analyzes gender differences, and explores health inequity. The major findings are as follows.

(1) This study uses the WHOQOL-BREF scale to measure the QOL of landless peasants. We use Analysis of Variance and Nonparametric Tests to test whether the difference of mean value of testing generals have statistical significances. the results shows significant differences occur between the impacts of different genders on the four domains of QOL (physical health, psychological health, social relationships, and environment). The use of Cronbach’s α coefficients indicates that the internal reliability of the scale is good, and the four domains of QOL are closely connected.

(2) Under the 100-score scale, scores in the four domains of QOL are relatively low, and those of females are much lower than those of males. Females generally obtain a lower QOL than males. Thus, Hypothesis 1 is supported.

(3) Results of the CFA analysis on each QOL domain and its related observed variables indicate good model fit, and the observed variables reflect each QOL domain.

(4) Estimation on the path coefficients of the gender differences analysis based on CFA shows that when others remain unchanged, scores in physical health increase with 1, and the QOL of both males and females increases with 1; when scores in psychological health increase with 1, the QOL of males and females increases with 1.254 and 1.177, respectively; scores in social relationships increase with 1, and the QOL of males and females increases with 1.230 and 1.098, respectively; and when scores in environment increase with 1, the QOL of males and females increases with 1.205 and 1.057, respectively. Each domain shows less impact on the QOL of females than males. Thus, Hypothesis 2 is supported.

***First***, the QOL of females is significantly and commonly worse than that of males. This result is partly consistent with previous studies. Health inequity in genders has been a long-term phenomenon in rural China [46,47]. Several indicators (education, income, and occupation) associated with health imply that females are more disadvantaged than males. In traditional villages, the man, as the headmaster of a family, takes on a physically demanding job. His income may provide the greatest support for the entire family. Thus, he has to deal with more pressure. Meanwhile, women are engaged in less physically demanding work. After the birth of their children, women are more likely to give up jobs and choose to become housewives at home. Thus, women face relatively less pressure compared with men. Moreover, previous studies have reported that men are more likely to smoke than women in rural and urban China [60-62]; the former are likewise more likely to drink alcohol than the latter [62,63]. Excessive smoking or drinking is considered unhealthy.

***Second***, the four domains show different impacts on the QOL of landless male and female peasants. Psychological health shows the greatest impact on the QOL of females. Several reasons may explain the lower QOL scores that females obtain.

1) Landless female peasants have a weaker sense of security. They live under unsatisfactory living conditions, are hampered by traffic conditions, suffer from inadequate health services, and have limited information from their external environment. Women require more from their living environment because a comfortable living environment contributes to a healthy life. When women lose land, their sense of security sharply decreases and their living environment becomes unsatisfactory, consequently negatively affecting their QOL. With regard to traffic, its unfavorable conditions (i.e., inconvenience, overcrowding) cause women, whether those stranded in villages or hovering at the edge of city, difficulty in going about their daily lives with comfort and ease. Moreover, most women require health services for gynecological diseases, pregnancy, and other conditions. However, when a realistic health environment does not fulfill the requirements, the QOL of landless female peasants is considerably reduced.

2) Women living in urban villages in China are less likely to communicate with others or obtain substantial help from their friends. Migration has brought changes in women’s reproductive behavior [64]. These women are likewise dissatisfied with their sexual lives. Social relationships include relationships with families, friends, workmates, and so on. Their relationships with families have two aspects. On the one hand, their husbands ought to have off-farm work because they have lost land. This situation reduces communication with their husbands, which adversely affects the quality of their social relationships. On the other hand, their sexual lives are diminished or become unsatisfactory due to the unfavorable living environment and the absence of husbands. Their fundamental physical needs are not fulfilled, thus decreasing their QOL.

3) “High mobility among rural–to–urban migrants is associated with increased sexual risk” [65]. A study further pointed out that both migrants and non-migrants are at risk of HIV infection; thus, intervention programs that aim to reduce sexual risk behaviors should cover both groups in rural China [66]. And the utilization of medical services helps to improve health [67]. With regard to relationships with friends of the same age, they provide insufficient help. Female friends cannot offer valid help because they are limited by their ability. Male friends provide less help in traditional villages. As for relationships with colleagues, many women do not have jobs. Thus, considering that women require more daily communication than men, poor social relationships have worsened the QOL of landless female peasants.

4) Women are doubtful about their capacity to carry out their daily activities and their working ability itself. They likewise lack adequate sleep, which depletes their energy. Although the rural labor market development in China has improved the welfare of women, women have not achieved parity with men [68]. Landless female peasants, regardless of their identity as “female” or “peasants,” always suffer from gender or employment discrimination, which significantly affects their psychological health. Their efforts are always ignored and seldom recognized. They tend to easily lose their self-esteem and their passion for life. Physical and psychological health influence and complement each other. Under depressing and burdensome conditions, landless female peasants experience a decline in their quality of sleep, which reduces their energy, ultimately trapping them in the vicious cycle of declining physical and psychological health.

5) Women may find life meaningless, lack enjoyment in life, and lose their daily concentration. Subjectively, the third point highlights the important impact of psychological health on physical health; hence, women lacking in physical passion tend to have a poor mental status. Objectively, the lack of recreational activities resulting from the backward economy and incomplete infrastructure in rural China causes boredom and physical deterioration. And phenomenon of "empty-nest" is common in rural China, which negatively affects the HQOL [69]. Moreover, as they used to be engaged in agricultural activities, a working environment of “facing the loess and backing the sky” has put women in poor shape. Their lack of awareness of fashion and self-care as well as rare consideration for ways to overcome their poor shape produces strong negative emotions [70].

The peasants’ loss of land is an inevitable phenomenon in economic and social development and urbanization. Landless peasants have lost natural resources to support their livelihood. When local governments pursue economic development and focus on their own performance, they sacrifice the interests of peasants in the process of land acquisition, compensation, and resettlement, causing a range of adverse social consequences. But the assistance policies of governments can help to improve the QOL [71,72]. The results in this study are alarming. The QOL of landless peasants is relatively poor, and that of female peasants is even poorer. Government intervention is thus urgently required.

We suggest several recommendations. ***First***, the government should improve the living conditions of landless peasants, including those concerning traffic, recreation, and other infrastructure, as well as beautify the surrounding environment. ***Second***, the government should promote the social relationships of landless peasants by providing training lessons on occupational knowledge and skills, creating a good atmosphere for communication, and facilitating their inclusion in urban society. ***Third***, with regard to improving the physical and psychological health of landless peasants, the government should increase the number of health care facilities and size of the personnel, and promote health knowledge to increase their awareness of disease prevention.

This study has several limitations. *First*, the study derived samples in relatively developed cities. However, the development of the YRD region is not completely balanced, and some cities are backward. These cities are undergoing rapid urbanization, and many peasants are landless. Future research may expand its scope by examining a larger scale of the YRD region. *Second*, this study only considers the gender differences in the QOL of Chinese landless peasants. Future research may explore the associations between QOL and demographic characteristics (e.g. age and education.) *Third*, landless peasants are a large, vulnerable group in China; thus, long-term concern for their QOL should be emphasized to address health inequity and adjust the policies and efforts of governments.

## Conclusion

Urbanization has accelerated in China, and large amounts of arable land have been transformed into urban land. Moreover, the number of landless peasants has continually increased. Peasants lose not only their land, but also a series of rights and interests related with land. Problems of landless peasants have been long-standing; however, only a few studies have examined their health or QOL. Based on these reality needs and to fill the gap in the literature, we use the WHOQOL-BREF scale to perform an analysis and modeling. In this manner, we assess the QOL of landless peasants in the YRD region, analyze gender differences, and explore health inequity. The major findings are as follows.

***First***, the scores in each QOL domain are commonly low, whereas the scores of females are much lower, indicating a poorer QOL than that of males. The scores reveal the pessimistic QOL condition of landless female peasants, whose problems are more significant than those of land male peasants. To solve these problems, more social attention and support should be provided to this vulnerable group. Moreover, appropriate and reasonable intervention should be provided in a timely fashion to help them achieve normal QOL conditions.

***Second***, the order of importance of four domains affecting QOL from high to low (psychological health, social relationships, environment, and physical health) is the same for landless male and female peasants, whereas the proportion of importance is different between genders.

The results of this study highlight the importance of government intervention to improve the QOL of Chinese landless peasants to reduce health inequity. Reducing health inequity between genders requires the promotion of social concern for Chinese landless female peasants. Thus, the government should provide a timely, appropriate, and reasonable intervention to help landless peasants achieve normal QOL conditions.

## Competing interests

The authors declare that they have no competing interests.

## Authors’ contributions

YL wrote and revised the manuscript, was responsible for the design of the study, and performed the statistical analysis. SL participated in the design of the study and in the statistical analysis. Both authors read and approved the final manuscript.
